# Myome, découverte fortuite ou métrorragie: qui dit mieux?

**DOI:** 10.11604/pamj.2021.38.388.20314

**Published:** 2021-04-20

**Authors:** Bénilde Marie-Ange Tiemtoré-Kambou, Adama Baguiya, Prosper David Lamien, Adjiratou Koama, Aischa Madina Napon, Yomboué Abel Bamouni, Ousséini Diallo, Adama Gnoumou, Cissé Rabiou

**Affiliations:** 1 Unité de Formation et de Recherche en Sciences de la Santé, Université Joseph Ki-Zerbo, Ouagadougou, Burkina Faso,; 2 Service d´Imagerie Médicale et Radiologie Interventionnelle du Centre Hospitalier Universitaire de Bogodogo, Ouagadougou, Burkina Faso,; 3 Institut de Recherche en Sciences de la Santé, Ouagadougou, Burkina Faso,; 4 Service de Radiologie du Centre Hospitalier Pédiatrique Charles de Gaulle, Ouagadougou, Burkina Faso,; 5 Service de Radiologie du Centre Hospitalier Universitaire Yalgado Ouédraogo, Ouagadougou, Burkina Faso,; 6 Unité d´imagerie médicale, Clinique Yati, Ouagadougou Burkina Faso

**Keywords:** Fibrome, échographie, découverte fortuite, métrorragie, Ultrasound, fibroid, fortuitous discovery, metrorrhagia

## Abstract

**Introduction:**

le myome est une pathologie fréquente dans notre contexte, découverte de façon fortuite ou par des métrorragies; notre objectif était de mesurer l´association entre la taille des myomes et leur circonstance de découverte, et entre le siège des myomes et la survenue de métrorragies.

**Méthodes:**

nous avons mené une étude transversale de 6 ans de janvier 2012 à décembre 2018 incluant les femmes de 18 ans et plus reçues au Centre Hospitalier Universitaire (CHU) Bogodogo pour une échographie pelvienne ou abdomino pelvienne, chez qui au moins un myome utérin a été découvert. L'analyse a consisté en une régression logistique binaire pour la métrorragie et multinomiale pour les circonstances de découverte et la taille.

**Résultats:**

nous avons analysé 1049 femmes, chez qui 2294 myomes ont été répertorié par échographie: soit 2 myomes par femme. L´âge moyen était de 37 ans. Les femmes dont le plus gros myome était supérieur à 50mm représentaient 29,7% (n=311). Il existait une forte association entre le siège interstitiel, sous-séreux ou sous-muqueux et la survenue de métrorragies (p<0,001). La taille inférieure à 50mm était significativement associée à une découverte fortuite (p=0,016), mais pas à une métrorragie révélatrice (p=0,084). Les femmes qui avaient des myomes sous-muqueux (OR=3,13; IC95%= [1,45-6,76]), interstitiel et sous-muqueux (OR=2,24; IC95%= [1,05-4,78] et interstitiel, sous-séreux et sous muqueux (OR=3,57; IC95%= [1,88-6,76]) avaient une côte de présenter une métrorragie plus élevée. Les myomes inférieurs à 50mm avaient un rapport de cote deux fois plus élevé de se révéler de façon fortuite (RRR=1,80; IC95%= [1,25-2,62]) ou par des métrorragies (RRR=1,75; IC95%= [1,04-2,95].

**Conclusion:**

les associations de siège des myomes sont plus à risque de métrorragie.

## Abstract (en)

**Introduction:**

myoma is commonly diagnosed in our hospital. It can be accidentally discovered or discovered due to metrorrhagia. The purpose of this study is to evaluate the association between the size of myomas and the circumstances under which they are detected and between the location of myomas and the occurrence of bleeding.

**Methods:**

we conducted a cross-sectional study including women aged 18 or older undergoing ultrasound at the University Teaching Hospital Bogodogo and who were diagnosed with at least one uterine myoma over a period of 6 years from January 2012 to December 2018. Binary logistic regression was used to assess metrorrhagia while multinomial logistic regression was used to assess circumstances under which they were detected and size.

**Results:**

we assessed 1049 women, among whom 2294 had myomas diagnosed on ultrasound. Each woman had two myomas. The average age of patients was 37 years. Women with myomas larger than 50 mm accounted for 29.7% (n=311). There was a strong association between interstitial, subserosal and submucosal myomas and the occurrence of metrorrhagias (p<0.001). A size less than 50 mm was significantly associated with fortuitous discovery (p=0.016) but not with revealing metrorrhagia (p=0.084). Women who had submucosal myomas (OR=3.13; CI95%= [1.45-6.76]), interstitial and submucosal myomas (OR=2.24; CI95%= [1.05-4.78] as well as interstitial, subserosal and submucosal myomas (OR=3.57; CI95%= [1.88-6.76]) were at higher risk of developing metrorrhagia. Myomas measuring less than 50 mm had twice the odds of revealing fortuitously (RRR=1.80; CI95%= [1.25-2.62]) or by metrorrhagia (RRR=1.75; CI95%= [1.04-2.95].

**Conclusion:**

metrorrhagia is more common in women with myomas in specific locations.

## Introduction

Les léiomyomes utérins ou les fibromes utérins sont les tumeurs gynécologiques les plus courantes et surviennent chez environ 20 à 50% des femmes dans le monde [1]. L'échographie est l'examen d'imagerie de première intention pour les fibromes suspectés et montre une sensibilité et une spécificité élevées dans le diagnostic de cette affection [1, 2]. Que le léiomyome soit symptomatique ou non dépend principalement de sa taille et de son emplacement [3]. Ce sont les tumeurs bénignes développées au dépens des cellules musculaires de l´utérus, œstrogéno-dépendantes. L´incidence des fibromes augmente avec l´âge: 20 à 30% des femmes caucasiennes et près de 50% des femmes de race noire de plus de 30 ans ont des fibro-myomes utérins [4]. Leurs manifestations cliniques sont multiples, toutefois la majorité d´entre eux est asymptomatique [5]. Ils sont asymptomatiques et découverts lors de complications à type de douleurs, de pesanteur, de métrorragies ou fortuitement lors de désir de maternité [6]. Ces circonstances de découvertes peuvent parfois être liées à leur siège. L´objectif de notre étude était de mesurer l´association entre la taille des myomes et leur mode (ou sa circonstance) de découverte et de mesurer l´association entre le siège des myomes (muqueux ou non) et la survenue de métrorragies.

## Méthodes

Il s´est agi d´une étude transversale avec recueil rétrospectif des données de janvier 2012 au 31 décembre 2018. La population d´étude était constituée de toutes les femmes de 18 ans et plus reçues au CHU de Bogodogo pour une échographie pelvienne ou abdomino-pelvienne, chez qui nous avons découvert au moins un myome utérin. Les échographies ont été réalisées avec un appareil Siemens A 300 mis en service en mars 2017 et un appareil Mindray Expert DC20 mis en service en mars 2012.

Les examens ont été réalisés par quatre médecins radiologues de 10, 5 et 2 ans d´expérience. La voie d´abord était sus pubienne plus ou moins complétée par la voie endovaginale en fonction des indications et de la bonne échogénicité de la patiente. La collecte a été faite à l´aide des comptes rendus d´échographie. Les variables étudiées étaient l´âge des patientes, les indications des échographies, les sièges des myomes, le nombre de myomes et la taille des myomes. Les données ont été saisies dans la base de données Microsoft Excel et analysées à l´aide du logiciel Stata 15.0 pour Windows. L´analyse a consisté en une régression logistique binaire pour la métrorragie et une régression logistique multinomiale pour les circonstances de découverte et la taille.

## Résultats

**Figure 1:**
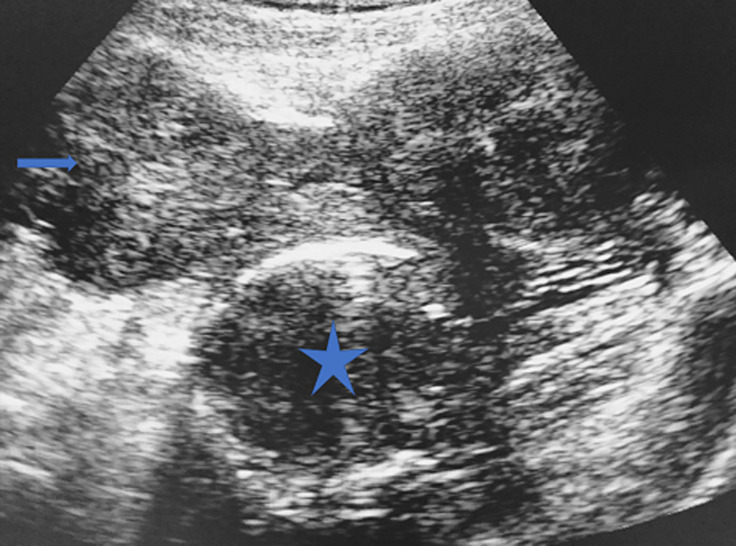
utérus déformé (coupe échographique transversale par voie suspubienne utérus polymyomateux à contours irréguliers chez une patiente de 32 ans avec une masse pelvienne, myomes interstitiels antérieurs refoulant la ligne cavitaire, myome sous séreux postérieur à paroi calcifiée)

Sur les 8260 patients de la période d´étude ayant eu à effectuer une échographie, nous avons analysé au total, 1049 femmes soit 12,69%, chez qui nous avons répertorié 2294 myomes: soit en moyenne 2 myomes par femme. L´âge moyen des femmes étaient de 37 ans avec un écart-type de 8 ans. La tranche d´âge la plus représentée était de 30-45 ans à 65,19% (663). Les moins de 30 ans et les plus de 45 ans étaient respectivement représentées à 16,91% (172) et 17,90% (182). Le plus jeune âge était de 18 ans. La grossesse était associée aux myomes dans 13,35% des cas (130). L´utérus était déformé dans 21,73% soit 228 cas (Figure 1). Le nombre moyen de myomes était de 2 [1;10]. Concernant le nombre de myomes par femmes, 39,94% (419) de la population d'étude avait 1 myome, 28,60% (300) avait 2 myomes, 16,59% (174) avait 3 myomes, 8,20% (86) femmes avaient 4 myomes et 70 femmes soit 6,67% avait 5 myomes à 10 myomes.

Les myomes étaient découverts de façon fortuite dans 34,70% (364) des cas, par des métrorragies dans 12,11% (127) cas et par les autres signes dans 46,33 (486) cas. Les circonstances n´étaient pas précisées dans 6,86% (72) cas. Les myomes symptomatiques étaient de 58,43% dont 12,11% révélées par des métrorragies. La majorité des femmes avait des myomes interstitiels (48,9%, n=509). La répartition des myomes selon leur siège interstitiel, sous-séreux ou sous-muqueux se présente dans le Tableau 1. La plupart des femmes avaient des myomes corporéaux dans 61,8% (572) cas, puis des myomes fundiques à 13,6% (124), les myomes corporéo isthmiques 13,2% (122) et des myomes isthmiques dans 11,4% (105). Les femmes dont le plus gros myome était supérieur à 50mm représentaient 38,12% (860) et celles dont le plus gros était inférieur à 50mm représentaient 1396. La taille du plus gros myome était 551mm. De façon générale, la taille médiane des myomes était de 32mm [21;48].

**Tableau 1:** répartition des femmes selon le siège des myomes

Siège des myomes	Effectif	Pourcentage
Interstitiel	509	48,9
Sous-séreux	123	11,8
Sous-muqueux	67	6,4
Interstitiel et sous-séreux	166	16,0
Interstitiel et sous-muqueux	68	6,5
Sous-séreux et sous-muqueux	33	3,2
Interstitiel, sous-séreux et sous-muqueux	75	7,2
Total	1041	100

**Figure 2:**
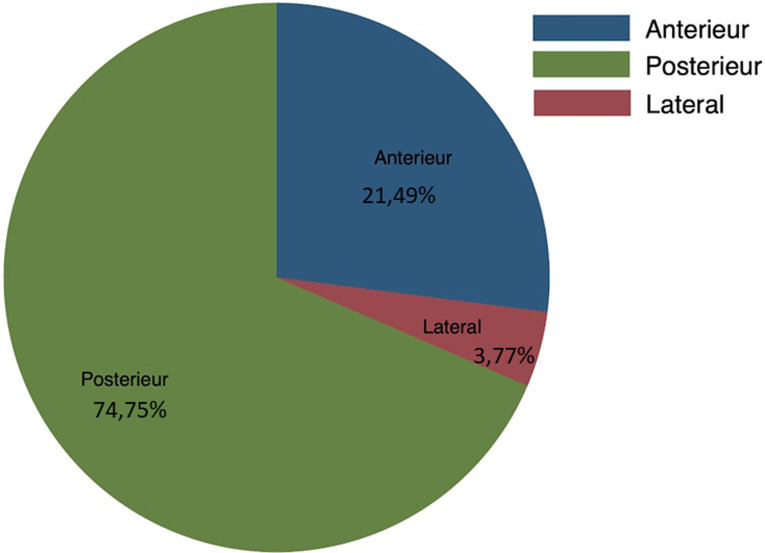
répartition des myomes en fonction du siège utérin

Les femmes avaient surtout des myomes postérieurs (68,4%, n=584) et les myomes latéraux étaient les moins nombreux (4,4%, n=38). La Figure 2 montre la répartition des myomes selon la position. Le Tableau 2 représente la relation entre les caractéristiques et les circonstances de découverte en analyse bi variée. Le Tableau 3 illustre la relation entre les circonstances de découverte et les variables âge, nombre de myome, taille du myome, siège du myome en analyse multivariée. Concernant la relation entre le siège des myomes et la survenue de métrorragie, il y avait 15 myomes sous muqueux associés aux métrorragies. En analyse bi-variée, il existait une forte association entre le siège interstitiel, sous-séreux ou sous-muqueux et la survenue de métrorragies (p<0,001). En ajustant sur l´âge de la femme, la position et la taille du myome, les femmes qui avaient des myomes sous-muqueux (OR=3,13; IC95%= [1,45-6,76]), interstitiel et sous-muqueux (OR=2,24; IC95%= [1,05-4,78]) et interstitiel, sous-séreux et sous muqueux (OR=3,57; IC95%= [1,88-6,76]) avaient une côte de présenter une métrorragie plus élevée que celle des femmes qui n´avaient que des myomes interstitiels. Ces données sont représentées dans le Tableau 4. Quant à la relation entre la taille des myomes et les circonstances de découverte, les 15 myomes sous muqueux associés aux métrorragies avaient une médiane de taille de 44mm avec un espace inter-quartile de 19-54. Les 197 myomes interstitiels découverts de façon fortuite avaient une taille moyenne de 40,5mm +/- 25mm.

**Tableau 2:** relation entre les caractéristiques et les circonstances de découverte en analyse bi variée

Variables	Découverte fortuite		Métrorragies		Autres signes		P
Siège	n	%	n	%	n	%	
Interstitiel	197	41,56	52	10,97	225	47,47	0,00
Sous séreux	47	41,59	13	11,50	53	46,90	
Sous muqueux	16	25,40	15	23,81	32	50,79	
Interstitiel et sous séreux	50	32,26	9	5,81	96	61,94	
Interstitiel et sous muqueux	26	41,27	13	20,63	24	38,10	
Sous séreux et sous muqueux	10	32,26	6	19,35	15	48,39	
Interstitiel, sous séreux et sous muqueux	16	22,54	19	26,76	36	50,70	
**Age**							
Moins 30 ans	73	44,8	19	11,7	71	43,6	0,00
30 à 35 ans	253	40,9	78	12,6	288	46,5	
45 ans et Plus	29	17,1	25	14,7	116	68,2	
**Deformation de l'utérus**							
Oui	72	93,3	37	17,1	107	49,5	0,091
Non	292	38,4	90	11,8	379	49,8	
**Nombre de myomes**							
1	159	40,7	59	15,1	173	44,2	0,006
2	115	42	27	9,8	132	48,9	
3	53	31,9	23	13,9	90	54,2	
4	20	25,6	11	14,1	47	60,3	
5 et plus	17	25	7	10,3	44	64,7	
**Grossesse**							
Oui	83	64,8	21	16,4	24	18,8	0,000
Non	281	33,1	106	12,5	462	54,4	
**Taille du plus grand myome**							
Inf 50 mm	256	39,6	89	13,8	301	46,6	0,012
Sup 50 mm	103	32,3	35	11	181	56,7	
**Position du myome**							
Antérieur	84	38,9	29	13,4	103	47,7	0,478
Latéral	18	47,4	2	5,3	18	47,7	
Postérieur	197	36,4	77	14,2	268	49,5	

**Tableau 3:** relation entre les circonstances de découverte et les variables âge, nombre de myome, taille du myome, siège du myome en analyse multivariée

	Découverte fortuite				Découverte à la suite de métrorragies			
Variables	RRR	95%CI		p	RRR	95%CI		p
**Age**								
30 à 45 ans	0,97	0,64	1,46	0,882	1,06	0,56	1,99	0,861
45 ans et plus	0,30	0,17	0,55	0,000	1,07	0,51	2,26	0,859
**Siège**								
Sous sereux	0,74	0,45	1,24	0,256	0,71	0,31	1,64	0,419
Sous muqueux	0,35	0,16	0,77	0,009	2,13	1,01	4,50	0,047
Interstitiel et sous-séreux	0,77	0,48	1,24	0,281	0,65	0,29	1,50	0,314
Interstitiel et sous-muqueux	1,73	0,88	3,41	0,113	2,88	1,21	6,86	0,017
Sous-séreux et sous-muqueux	0,78	0,32	1,89	0,583	2,01	0,64	6,27	0,230
Interstitiel, sous-séreux et sous-muqueux	0,48	0,22	1,07	0,072	3,73	1,69	8,24	0,001
**Nombre de myomes**								
2	0,85	0,56	1,30	0,457	0,66	0,35	1,24	0,198
3	0,61	0,37	1,02	0,062	0,51	0,24	1,08	0,079
4	0,37	0,19	0,73	0,004	0,50	0,20	1,25	0,137
77	0,34	0,16	0,72	0,005	0,40	0,14	1,14	0,086
**Taille du plus grand myome**								
50 mm et plus	0,87	0,61	1,23	0,422	0,82	0,50	1,33	0,420
**Segment**								
Corporéal	1,09	0,69	1,72	0,718	1,00	0,51	1,97	0,996
Isthmique	1,47	0,77	2,78	0,240	0,81	0,28	2,33	0,701
Corporéo-isthmique	1,12	0,60	2,09	0,731	1,21	0,51	2,91	0,667

**Tableau 4:** relation entre siège des myomes et la survenue de métrorragies

Variable	Odds ratio	Intervalle de confiance à 95%		P value
**Siège**				
Interstitiel	Réf			
Sous-séreux	0,79	0,31	2,02	0,627
Sous muqueux	3,13	1,45	6,76	0,004
Interstitiel et sous-séreux	0,66	0,31	1,41	0,284
Interstitiel et sous-muqueux	2,24	1,05	4,78	0,037
Sous-séreux et sous muqueux	1,90	0,67	5,39	0,230
Interstitiel, sous-séreux et sous muqueux	3,57	1,88	6,76	0,000
**Age**	0,99	0,97	1,02	0,673
**Position**				
Antérieure	Réf.			
Latérale	0,41	0,09	1,84	0,244
Postérieure	0,76	0,46	1,26	0,287
**Myome inférieur à 50 mm**				
Non	Réf			
Oui	1,36	0,83	2,23	0,222

**Figure 3:**
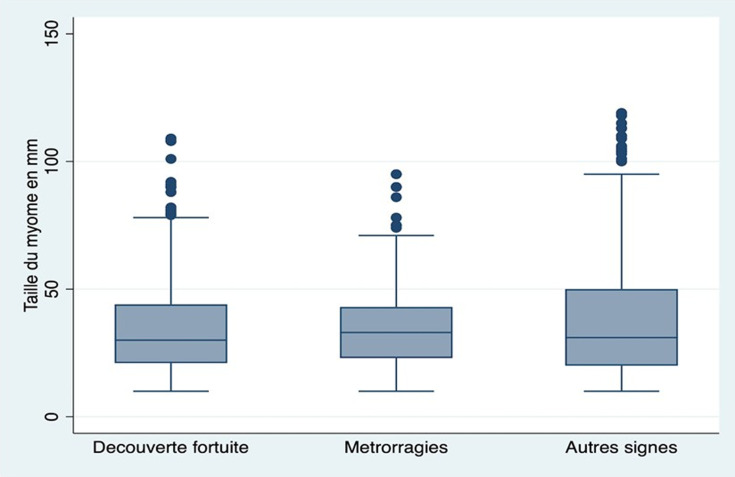
relation entre taille des myomes et circonstance de découverte

En analyse bi variée, la taille de myome inférieure à 50mm était significativement associée à une découverte fortuite (p=0,016), mais pas à une métrorragie révélatrice (p=0,084), par rapport à une découverte par les autres signes fonctionnels. La Figure 3 présente les statistiques descriptives de la taille des myomes selon la circonstance de découverte. Le Tableau 4 illustre l´analyse multivariée, après ajustement sur l´âge de la femme, le siège et la position du myome. Nous avons trouvé que par rapport à une découverte par les autres signes fonctionnels, les myomes qui avaient une taille inférieure à 50mm avaient un rapport de cote deux fois plus élevé de se révéler de façon fortuite (RRR=1,80; IC95%= [1,25-2,62]) ou par des métrorragies (RRR=1,75; IC95%= [1,04-2,95], par rapport à une découverte par d´autres signes fonctionnels.

## Discussion

Le nombre de femmes présentant des fibromes dans la population d´étude au cours de la période était de 12,69%. Ce taux correspond à la proportion retrouvée dans la littérature de 5,7 à 77%, [7, 8]. Les léiomyomes utérins ou les fibromes utérins sont les tumeurs gynécologiques les plus courantes et surviennent chez environ 20 à 50% des femmes dans le monde [9]. Les différences raciales pointent du doigt la race noire et le taux retrouvé est corroboré par Laughin *et al*. [10] qui trouve une prévalence de 18% chez les Noires, 8% chez les femmes Blanches, 10% chez les hispaniques et 13% chez les autres largement constituées par les Asiatiques. L'échographie (USG) est l'examen d'imagerie de première intention pour les fibromes suspectés et montre une sensibilité et une spécificité élevées dans le diagnostic de cette affection [2]. Un biais de sélection pourrait expliquer ce taux car c´est une population hospitalière. Cela pourrait aussi être sous-estimée par l´abord abdominal pas toujours corrélée à la voie endovaginale même pour les petits fibromes, bien que la littérature ait montré la supériorité de la voie vaginale par rapport à celle abdominale [1].

L´âge des patientes était sensiblement comparable à celui de Massoud en Algérie avec une moyenne de 33 ans et un plus jeune âge de 18 ans comme dans notre étude [11]. Geum Seon Sohn trouve que plus on se rapproche de la ménopause surtout vers la quarantaine plus les myomes sont fréquents [12]. Le groupe des 30-45 ans était le plus représenté à 663 (65,19%) et celui des plus de 45 ans à 182 (17,90%) femmes. Les myomes symptomatiques étaient de 58,43% dont 12,11% révélées par des métrorragies. Ces résultats sont corroborés par Ardaens chez qui 20 à 50% des fibromes sont symptomatiques [13]. Les myomes interstitiels étaient les plus nombreux chez 48,9% (n=509) de la population. La prépondérance des myomes intramuros est unanimement reconnue par Zeghal, Murase et Bilé-Gui [14-16]. Le siège corporéal prépondérant est également retrouvé chez Massoud [11]. Ensuite vient le siège isthmique alors que dans notre étude le siège isthmique venait en troisième position précédé du siège fundique.

Le nombre de myome par patiente était semblable à celui de Monleóna *et al*. [17] entre 1 et 3 myomes mais inférieur à celui de Bilé-Gui de 9 [16]. L´âge moyen des patientes de notre étude (36,72 ans) qui correspond à celui des études caucasiennes de 35 et 37 ans [18, 19] et la voie d´abord sus pubienne occultant parfois certains myomes pourraient justifier ce résultat. Lorsque les patientes sont symptomatiques, le nombre, la taille et / ou la localisation des fibromes sont des déterminants critiques de leurs manifestations cliniques. Les symptômes les plus fréquemment rapportés comprennent des saignements menstruels abondants, une dysménorrhée, des douleurs non cycliques, des symptômes urinaires, de la fatigue et de la constipation [3, 11, 20]. Une méta-analyse récente a démontré que les fibromes sous-muqueux, intra-muros et sous-séreux ont des effets différents sur la fertilité et qu'ils sont principalement liés à des lésions sous-muqueuses entraînant des défauts d'implantation [12].

Dans notre étude l´association interstitiel, sous séreuse et sous muqueuse avait quatre fois plus de risque d´être découverte par des métrorragies avec un p significatif. Cela pourrait être dû au fait que ces fibromes sont souvent associés à une hyperplasie de l´endomètre, ils induisent également des troubles locaux de la crase sanguine et les fibromes sous-muqueux sont la variété de fibrome principalement responsables de saignements par altération de l´endomètre ainsi que des mécanismes physiologiques des règles [21, 22] Ceux interstitiels et sous muqueux avaient 3 fois plus de risque d´être découverts par une métrorragie, comme si plus il y avait d´association de myomes dans les différentes tuniques de l´utérus, plus le risque de métrorragie était élevé. Selon Cravello [23] les myomes interstitiels entraînent des ménorragies par hyperplasie endométriale associée dans un contexte d´insuffisance lutéale. Les myomes sous-muqueux intra-cavitaires eux sont particulièrement hémorragiques, même lorsqu´ils sont de volume modéré [21]. En effet dans notre étude les 15 myomes sous muqueux associés aux métrorragies avaient une taille médiane de 44mm qui est un volume modéré.

La découverte fortuite se retrouve aussi dans le siège sous muqueux avec p significatif. La taille inférieure à 50mm avait un rapport de cote de se révéler de façon fortuite deux fois plus comme par une métrorragie avec un p significatif. En effet dans notre contexte les 197 myomes interstitiels découverts de façon fortuite était de 40,5mm Cette taille ne dépassant pas la tunique utérine ne pouvait être révélée que par les moyens en imagerie et ici l´échographie occupe toute sa place en permettant de façon accessible le diagnostic de ces fibromes. Par contre cette taille inférieure à 50mm découverte par des métrorragies est inhabituelle; serait-elle associée à d´autres facteurs?

## Conclusion

La découverte fortuite est fortement corrélée à la taille et au siège des myomes en interstitiel et en deçà de 5cm. Les métrorragies sont elles aussi corrélées au siège sous muqueux. Un aspect prospectif pourrait donner plus de poids à ces assertions vu la fréquence de cette pathologie dans notre contexte. Les associations myomateuses sont plus à risque de découverte par des métrorragies. Toutefois certains myomes sous muqueux ont été découverts de façon fortuite. Cela donnerait une orientation pour la prise en charge des fibromes qui est toujours d´actualité.

### Etat des connaissances sur le sujet

- Les myomes sont fréquents;
- Les myomes de moins de 10cm sont la plupart du temps découverts de façon fortuite;
- Les métrorragies sont les circonstances de découverte des myomes sous muqueux.

### Contribution de notre étude à la connaissance

- Les myomes intra-cavitaires sous muqueux peuvent être découverts de façon fortuite;
- L´association des sièges (sous muqueux, sous séreux et interstitiels) des myomes est plus pourvoyeuse de métrorragie;
- Les myomes qui avaient une taille inférieure à 50mm avaient un rapport de cote deux fois plus élevé de se révéler de façon fortuite.
